# Differential inhibition of gelatinase activity in human colon adenocarcinoma cells by *Aloe vera* and *Aloe arborescens* extracts

**DOI:** 10.1186/s12906-020-03134-9

**Published:** 2020-12-12

**Authors:** Ana Lima, Paula Batista-Santos, Eduarda Veríssimo, Patrícia Rebelo, Ricardo Boavida Ferreira

**Affiliations:** Plants for Health and Nutrition, LEAF (Linking Landscape, Environment, Agriculture and Food), Instituto Superior de Agronomia, Universidade de Lisboa, Tapada da Ajuda, 1349-017 Lisbon, Portugal

**Keywords:** Aloe, Colon cancer, Inflammation, Gelatinases, Extraction

## Abstract

**Background:**

*Aloe*’s reported bioactivities (anticancer, anti-inflammatory and wound healing) suggest they might inhibit a subgroup of matrix metalloproteinases (MMPs) called gelatinases (MMP-2 and MMP-9). The goal of the present study was to compare the MMP inhibitory potential of two *Aloe* species, *A. vera* and *A. arborescens*.

**Methods:**

Different types of extraction were tested and specific bioactive compounds were quantified. Cancer cell invasion inhibitory activities were measured in vitro using the wound healing assay in human colon cancer cells (HT29). Effects on gelatinase activities were further assessed by dye-quenched gelatin and gelatin zymography.

**Results:**

Different types of extraction yielded significantly different levels of bioactivities and of bioactive compounds, which might be due to a greater amount of extractable bioactive compounds such as anthraquinones. Both *A. arborescens* and *A. vera* have potential as inhibitory agents in cancer cell proliferation via MMP-9 and MMP-2 enzymatic activity inhibition, being able to reduce colon cancer cell proliferation and migration but *A. arborescens* showed to be a more effective inhibitor of cancer cell migration than *A. vera*.

**Conclusion:**

This work opens novel perspectives on the mode of action of *Aloe* species in cancer cell migration and may provide clues as to why there are so many conflicting results on *Aloe*’s activities.

## Background

The plant genus *Aloe* is largely known for its medicinal purposes for many centuries. Its uses are mentioned to as early as in Egyptian papyri, the Dioscorides’ Materia Medica (~ 60 CE) or the Garcia de Orta’s Colloquies (1563 CE in Goa) [1, 2]. Presently, Aloe species account among the most economically important medicinal plants and are commonly used for several ailments, namely anti-inflammatory, antiulcer, and immunomodulatory to antimicrobial activities [3–6]. Several studies have revealed the effectiveness of *Aloe* plants towards various types of cancer, like colon, duodenal, intestinal, lung, kidney pancreatic, liver, and skin, and these works have been confirmed through numerous in vitro and in vivo experiments [7, 8].

Nonetheless, antagonist results arise frequently: whilst many papers described its anticancer properties [7, 8], others surprisingly claim *A. vera* is indeed carcinogenic over a specific dose [9] or alert that specific compounds present in *Aloe* may be highly toxic if not taken under caution [10]. One reason for this could be the overall misidentification of the *Aloe* genus species, since current research supports that there are *Aloe* species that have more potent bioactivities than *Aloe vera* [11].

Today, *A. barbadensis* Mill. (*A. vera*) is incontestably the most common species of *Aloe* used worldwide, often incorrectly mentioned to as the one with the highest bioactivities in anecdotal reports [12, 13]. Yet, there are about 500 *Aloe* species documented, from which just a small fraction has reported ethnomedicinal uses [11]. Additionally, the phenotype similarities among species are enormously high, inducing people to use frequently the wrong species. Hence, because of its huge medicinal, cosmetic and other demands there is a high possibility that some species may often be mistakenly identified for different medicinal usages [14]. On the other hand, the use of single specific phytochemical derived from *Aloe* instead of the crude and/or standardized extracts could affect the overall bioactivities since it has been shown that many components in *Aloe* act synergistically with the remaining constituents in the plant [14]. In this sense, it also noteworthy to state that there is an overall lack of consistency regarding the type of extractions to use. Several works report the use of aqueous extracts [15] whilst others mention to use alcoholic or organic solvents like acetone or methanol [16, 17]. Taking into account that *Aloe*’s main phytochemical groups vary from phenols to proteins, carbohydrates, vitamins and organic and inorganic compounds [3], it is relevant to explore different types of extractions.

Another important factor to consider is the method of functioning of this genus’ bioactivities. Currently, a particular group of matrix metalloproteinases (MMPs) named gelatinases (MMP-2 and MMP-9) is greatly associated to cancer invasion through degradation of the cellular matrix, and the consequent release of cancer cells via proteolysis [18–20]. Since MMP-9 activities have also been closely associated to inflammation and delay in wound closure [21, 22] it is very likely that *Aloe* compounds could target these gelatinases. Indeed, concerning inflammation, *Aloe vera* extracts are recognized to down regulate metalloproteinase expression, precisely MMP-9, which contributes to the extracellular matrix degradation when recruited by cytokines to inflammation sites [23]. Hence, it is quite probable that *Aloe* species might inhibit enzymatic activity of MMP-2 (72 kDa) and MMP-9 (92 kDa) in cancer-related situations. However, to our knowledge there has been no link established between gelatinase activity inhibition induced by *Aloe* plants and cancer.

These studies can be valuable to confirm the real effect of *Aloe* species on cancer and eventually provide the identification of innovative anticancer approaches. Furthermore, the analysis of gelatinase activity inhibition by *A. vera* extracts may also constitute a novel perspective, which can help to comprehend the mechanisms of action of the plants from this genus. With that in mind, we set out to evaluate the content of several classes of potentially bioactive compounds, using different extraction procedures and their impact on the growth and migration of colon adenocarcinoma cells, as well as their effect on MMP-9 and MMP-2 activities.

## Methods

### Species selected and collection of plant samples

Leaves of *A. vera* and *A. arborescens* were collected from the existing Collection of *Aloe* genus specimen of Parque Botânico da Tapada da Ajuda (member of the Botanic Gardens Conservation International – BGCI), Instituto Superior de Agronomia, University of Lisbon (ISA/UL), Portugal. The species were kindly identified by Dr. Nuno Costa, Botanist, and are still alive in Parque Botânico da Tapada da Ajuda, ISA/UL, Portugal. All selected individuals had several years of existence and were not in the flowering season. Leaf samples were collected from at least three different plants.

### Preparation of the leaf extracts

Fresh leaves were washed with distilled water and chopped into small fragments of approximately 20 g each. Three different extraction methods were performed using different solvents at a ratio of 1:5 (w/v): 1) 100% (v/v) methanol, 2) 50% (v/v) methanol, and 3) 100 mM Tris-HCl buffer, pH 7.0. All extractions were performed by grinding a sample of 20 g with the respective solvent using an ULTRA-TURRAX T25 (IKA®Labortechnik) grinder, followed by agitation for 4 h at 4 °C. Both extracts containing methanol were evaporated in a bath at 60 °C (Kottermann) whereas the aqueous extracts were desalted through filtration using 3 kDa membrane centricons and centrifuged at 2000 *g*. All extracts were then lyophilized (Edwards Modulyo EF4) for 24 h and the obtained powder was weighted and stored at − 20 °C.

### Quantification of potentially bioactive compounds

#### Proteins

Protein quantification was performed using the standard Bradford method as described by Bradford [24]. The samples were read in a spectrophotometer Syenery HT, Bio-TEK at 595 nm and bovine serum albumin was used as standard. All samples used in protein quantification were treated with PVPP to eliminate the phenolic compounds that could interfere with the extraction of soluble proteins [25].

#### Phenolic compounds

The phenolic compounds were quantified using the Folin-Ciocalteau reagent using gallic acid as standard. The lyophilized powder (corresponding to 20 g of fresh leaves) was treated with 10 μL of 70% (v/v) acetone, 10 μL of 0.5% (v/v) acetic acid and 80 μL of 7% (w/v) sodium carbonate. Subsequently, a volume of 100 μL of Folin-Ciocalteau was added to the solution and the mixture was vortexed. The solution of 200 μL was incubated for 8 min at room temperature and the absorbance was read in a Syenery HT Bio-TEK spectrophotometer at 765 nm [26].

#### Anthraquinone

To 300 μL of the previous extracts, 300 μL of pure benzene were added and the resulting solution incubated with agitation at 0 °C bath for 30 min. It was then centrifuged at 4.500 *g* for 20 min at 4 °C in a Beckman J2-21M/E centrifuge. To the recovered supernatant 500 μL of 10% (v/v) ammonia solution were added and the absorbance read at 515 nm using Aloe blue curacao aloin as a standard [27].

#### Total carbohydrates

Sugar quantification was performed utilizing the phenol/sulfuric acid method and mannose was used as the standard. To the previous extracts a 4% (v/v) phenol solution was added in a proportion of 1:5 and then incubated 5 min at room temperature. Afterwards, a 1:40 proportion of sulfuric acid was added and the absorbance read at 492 nm in the equipment mentioned above [28].

### In vitro colon cancer cell assays

The human colon adenocarcinoma cell line HT29 (ECACC 85061109) was purchased from the European Collection of Authenticated Cell Cultures, UK, and was used throughout this work. HT29 cell lines were maintained in RPMI medium supplemented with 10% (w/v) of heat-inactivated fetal bovine serum and 200 mM glutamine, 2 × 10^4^ UI/mL penicillin and 20 mg/mL streptomycin at 37 °C, in a humidified atmosphere of 5% (v/v) CO_2_.

### Cell proliferation assay

HT29 cultured cells were seeded on 96-well plates (2 × 10^4^ cells/well) and *Aloe* leaf extracts were added to the growth media to obtain different final concentrations, and incubated for 24 h. After each treatment, the extracellular media was collected, and the wells were washed with PBS to remove unattached cells. Cell proliferation and viability was determined using the standard 3-(4,5-dimethylthiazol-2-yl)-2,5-diphenyltetrazolium bromide (MTT) assay as described by Carmichael et al. [29].

### Cell migration assay

For cell migration analysis, the wound healing assay was performed. HT29 cells (5 × 10^5^ cells/well) were seeded in 6-well plates and allowed to reach to 80% confluence. Wounds were performed by making a scratch across the cell monolayer to create an open gap, mimicking a wound. Cells were then washed twice with PBS to remove floating debris. Each well was subsequently filled with fresh media containing the samples under study, in a concentration of 100 μg/mL and allowed to grow for 48 h. The invaded area after 48 h was calculated in each treatment and compared to the initial area at 0 h, to determine the area covered by migrating cells into the denuded zone at the beginning of treatment. This comparison allowed us to assess the inhibitory effect (if any) exerted by each protein fraction on the HT29 cell migrating capacity.

### MMP-9 and MMP-2 catalytic activities

#### Gelatinolytic activity with commercial MMPs

The fluorogenic substrate dye-quenched (DQ)-gelatin was purchased from Invitrogen (Carlsbad, CA, USA) and dissolved in water at 1 mg/mL. All solutions and dilutions were prepared in assay-buffer (50 mM Tris-HCl buffer, pH 7.6, containing 150 mM NaCl, 5 mM CaCl_2_ and 0.01% v/v Tween 20). A 96-well micro-assay plate (chimney, 96-well, black) was used. Each well was loaded with 0.1 mM (for a final volume of 200 μL) MMP-9 (Sigma), to which 100 μg/mL protein of total *Aloe* extract (for a final volume of 200 μL) was added, and the plate was incubated for 1 h at 37 °C. Subsequently, DQ-gelatin (at a final concentration of 2.5 μg/mL) was added to each well and the plate was incubated again, for 1 h. Fluorescence levels were measured (ex. 485 nm/em. 530 nm). In each experiment, both positive (no sample) and negative (no enzyme) controls were included for all samples, to correct for possible proteolytic activities present in the *Aloe* extracts. All data were corrected by subtraction of their corresponding negative controls.

#### Gelatinolytic activity with HT29 cell culture extracellular media

The same method described above was used, with some alterations. Roughly, each well was loaded with 100 μL of extracellular HT29 media (containing MMP-9 and MMP-2) after exposure to the *Aloe* extracts. Subsequently, DQ-gelatin (at a final concentration of 2.5 μg/mL) was added to each well (for a final volume of 200 μL) and the plate was incubated again, for 1 h. Fluorescence levels were measured (ex. 485 nm/em. 530 nm).

#### Gelatin zymography

To determine metalloproteinase activities in HT29 cancer cell culture supernatants, a gelatin-zymography was performed according to standard methods adapted by Lima et al. [30]. Cell culture supernatants were treated with 62.6 mM Tris-HCl buffer, pH 6.8, containing 2% w/v SDS, 10% v/v glycerol and 0.01% w/v bromophenol blue and separated in SDS-polyacrylamide gels (12.5% w/v acrylamide) with 1% (w/v) gelatin. After electrophoresis, gels were washed three times in 2.5% (v/v) Triton X-100 for 90 min each and incubated at 37 °C with developing buffer for 24 h. After staining with Coomassie Brilliant Blue G-250, the white bands visible marked the gelatinase activity of each proteinase. Protein band intensities were determined by densitometry as described earlier [30].

### Statistical analysis

All experiments were performed in triplicate, in at least three independent times and the data are expressed as the mean ± standard deviation (SD). SigmaPlot software (version 12.5) was used for comparing different treatments, using one-way and two-way analysis of variance (ANOVA). Tukey’s test was used to compare differences between groups and the statistical differences with *p* value less than 0.05 were considered statistically significant.

## Results

### The amount of bioactive compounds extracted from *A. arborescens* and *A. vera* is influenced by the species and by the extraction procedure

**Fig. 1:**
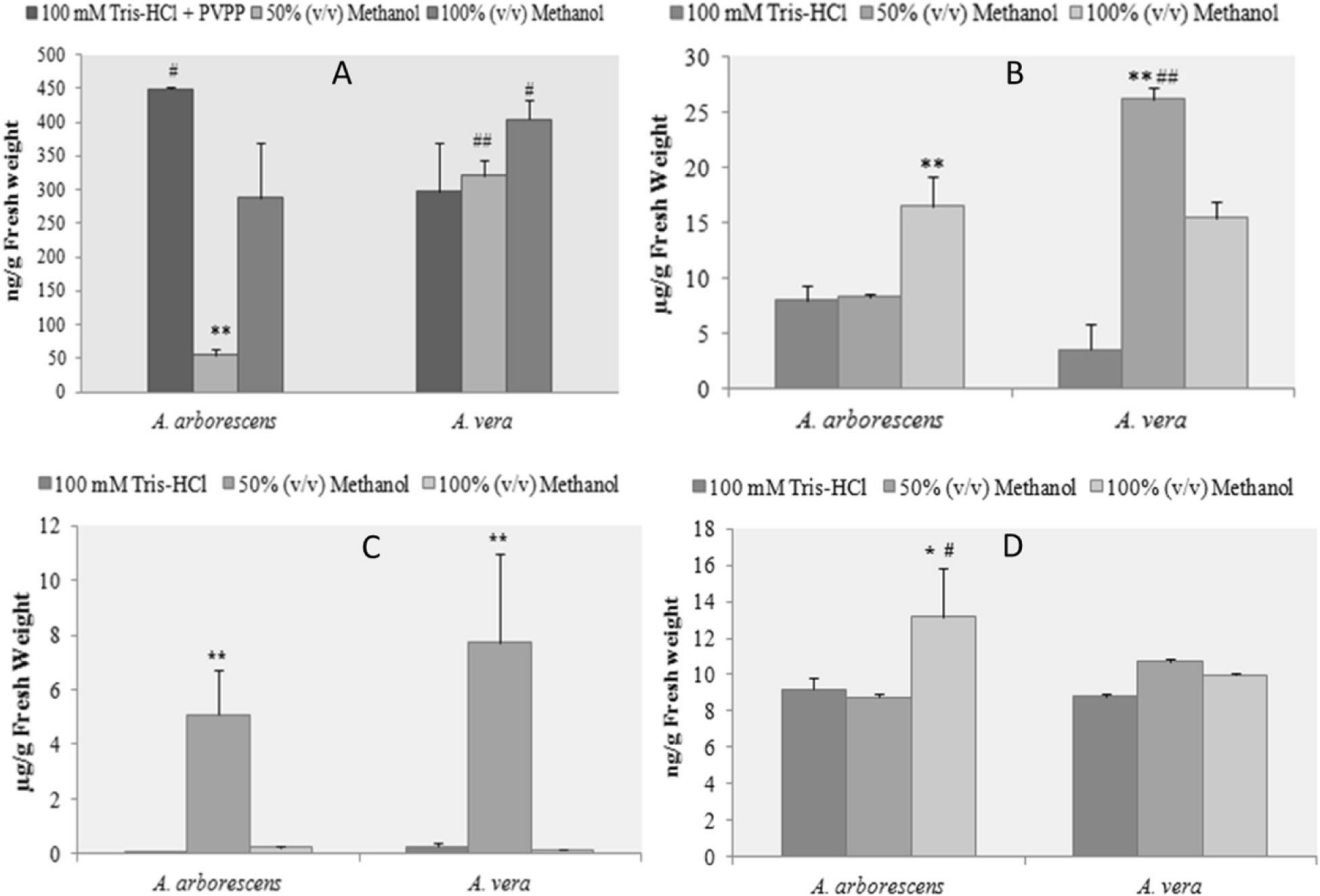
Quantitative characterization of *A. vera* and *A. arborescens* considering the main phytochemical groups defined by Hamman [3]. Each extraction was performed with 20 g of fresh *Aloe* leaves for each of the three solvents: 100 mM Tris-HCl buffer, 50% (v/v) methanol and 100% (v/v) methanol. **a** Protein quantification using the Bradford method described by Bradford (1976). All samples were treated with Polyvinylpolypyrrolidone (PVPP) to eliminate the phenolic compounds that could interfere with the extraction of soluble proteins. **b** Phenolic compounds quantification with the Folin-Ciocalteau reagent and using gallic acid as standard. **c** Total carbohydrates quantification using phenol/sulfuric acid method and mannose as the standard. **d** Specific anthraquinone quantification (phenol component) using benzene, ammonia solution and Aloe blue curacao aloin. * *p* < 0.05; **p < 0.001 when compared between extracts of the same species; # *p* < 0.05; ## *p* < 0.001 the same extract when compared between species

The amounts of the different classes of bioactive compounds in both *Aloe* species are present in Fig. 1. When comparing *A. vera* and *A. arborescens* we can easily identify differences between species regarding both the extraction methods for each component and the amount of each component per amount of fresh weight. Results presented in Fig. 1 (a) corroborate the low amount of proteins present in both species, which were overall less than 0.1% (w/v) of fresh weight. Nonetheless, protein amounts and distribution varied significantly between both species.

Whilst the amount of Tris-HCl-soluble protein was significantly higher (*p* < 0.05) in *A. arborescens* than in *A. vera*, *A. vera* presented the higher amounts of methanol-soluble proteins than *A. arborescens* (*p* < 0.05).

Phenolic compounds were already described as the second major class of compounds found in *A. vera* [31], second only to polysaccharides (Fig. 1 (b)). Regarding the results in Fig. 1 (b), corresponding to total phenols present per extraction, it is clear that the extraction with 50% (v/v) methanol yields higher amounts of total phenolic in *A. vera* extraction (26.19 μg/g), whereas for *A. arborescens* the extraction with 100% (v/v) methanol yielded the highest amounts (16.48 μg/g), in a significant manner (*p* < 0.05). Albeit in Fig. 1 (b) *A. vera* presented more phenolic compounds (*p* < 0.05) when compared to *A. arborescens*, when evaluating the specific group of anthraquinones (Fig. 1 (d)), *A. arborescens* had a significant higher content in the methanolic extractions.

Finally, regarding the composition in total polysaccharides between the two species (Fig. 1 (c)), the extraction with 50% (v/v) methanol presented a significantly higher yield than the other two extractions (*p* < 0.001). For example, *A. vera* contains 7.72 ng/g against 0.25 ng/g for 100 mM Tris-HCl buffer and 0.14 ng/g for 100% (v/v) methanol.

### Different Aloe extraction procedures exert different effects on cancer cell migration

**Fig. 2:**
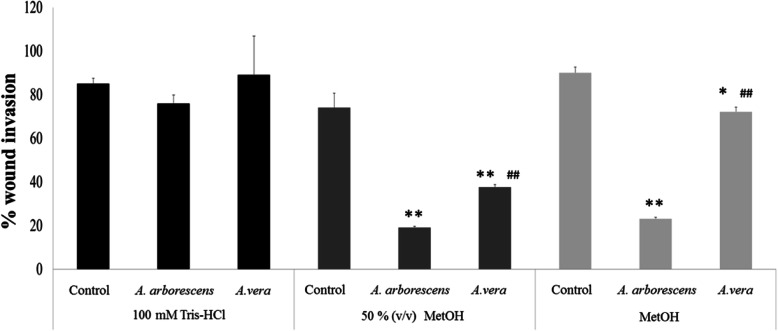
Percentage of cell migration after a 48 h period of HT29 cell exposure to *A. arborescens* and *A. vera* different extracts. Extracts were prepared with 100 mM Tris-HCl buffer, 50% or 100% (v/v) methanol (MetOH). * *p* < 0.05; **p < 0.001 when compared to controls on the same extraction; # *p* < 0.05; ## *p* < 0.001 when compared between the same species on the same type of extraction

To test and compare the anticancer and MMP-9 inhibitory potential of two extracts from *A. vera* and *A. arborescens*, a colon cancer cell line HT29 was selected, using the standard cell migration assays, and results are expressed in Fig. 2.

Since distinct extraction procedures seem to provide different quantities of various compounds and differ between *Aloe* species, we aimed to select the best extraction method for the HT29 cell assays, using buffer-soluble and methanolic extractions. The results expressed in Fig. 2 indicate that buffer-soluble extractions did not show any significant reduction in cell migration when compared to controls (*p* > 0.05), in contrast to the methanolic extractions where an overall reduction in cell migration was obtained by both species extracts. Concerning *A. arborescens*, both methanolic extractions led a significant reduction (*p* < 0.001) of about 80% in cell migration. In the case of *A. vera* the best results were obtained for 50% (v/v) methanolic extracts when compared to the 100% (v/v) methanolic extract (*p* < 0.05). As there was no difference for *A. arborescens* and as it provided the best results for *A. vera*, the 50% (v/v) methanolic extractions were selected for the remaining cancer cell assays.

### Aloe extracts influence HT29 colon cancer cell proliferation in a dose-dependent manner

**Fig. 3:**
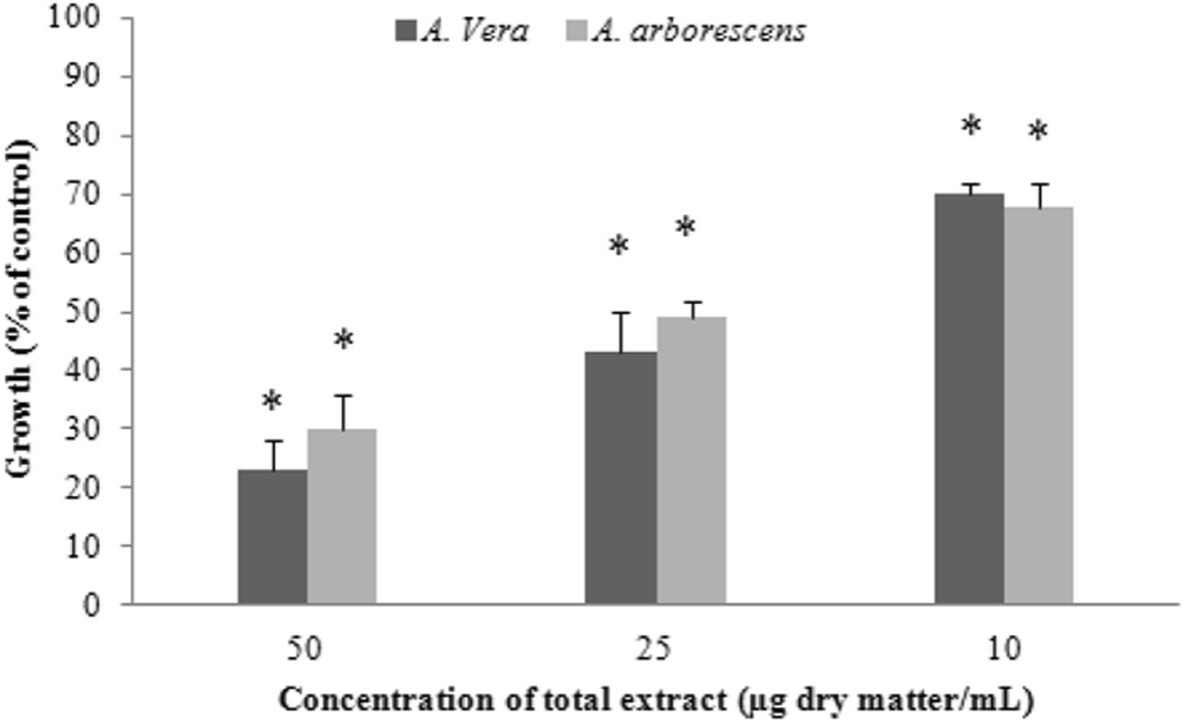
HT29 cell proliferation assay. Cells were exposed for 48 h to *A. arborescens* or *A. vera* extracts prepared with 50% (v/v) MetOH (methanol). Cell growth was obtained by the standard MTT assay. Results are expressed as the mean of at least three replicate experiments ±SD.*p < 0.05 between concentrations

To investigate whether the effect of the *Aloe* extracts inhibited colon cancer cell growth, we tested different concentrations of the selected 50% (v/v) methanolic *Aloe* extracts. Figure 3 shows the proportion of HT29 living cells after growth in the presence of 10, 25 and 50 μg dry weight/mL *Aloe* extracts, obtained after staining with MTT (which can only be metabolized by living cells). The results indicate that a 2-day exposure to the 50% (v/v) methanolic extract from both studied *Aloe* species did induce a significant reduction (*p* > 0.001) in cancer cell growth when compared to controls. Additionally, this reduction was highly dose-dependent, with higher concentrations leading a significantly higher inhibition than the previous (*p* < 0.05). Remarkably, even though it has been suggested that *A. vera* is the *Aloe* species with the lowest toxicity, our results indicate that there were no significant differences (*p* < 0.001) between *A. arborescens* and *A. vera* extracts for all the tested concentrations. Being dose-dependent, the results point to an EC50 around 25 μg dry weight/mL for the 50% (v/v) methanolic extracts (*A. vera*: 24.27 μg and *A. arborescens*: 26.8 μg).

The results for the minimal inhibitory concentrations (MIC), which in this work was determined as the lowest concentration which prevented 10% growth inhibition are presented in Table 1. MIC results point to much lower inhibitory concentrations, showing stronger inhibition for *A. arborescens*, with a MIC of 3.12 μg dry weight/mL, which is approximately half the MIC determined for *A. vera*.

**Table 1:** Minimal inhibitory concentrations for cell growth when HT29 cells were exposed to different concentrations of *A. vera* and *A. arborescens* extracts prepared with 50% (v/v) MetOH (methanol). Results are expressed in μg dry weight/mL

MICs (μg/mL)	
*A. vera*	6.25
*A. arborescens*	3.12

### EC50 Aloe extracts reduce colon cancer cell migration

**Fig. 4:**
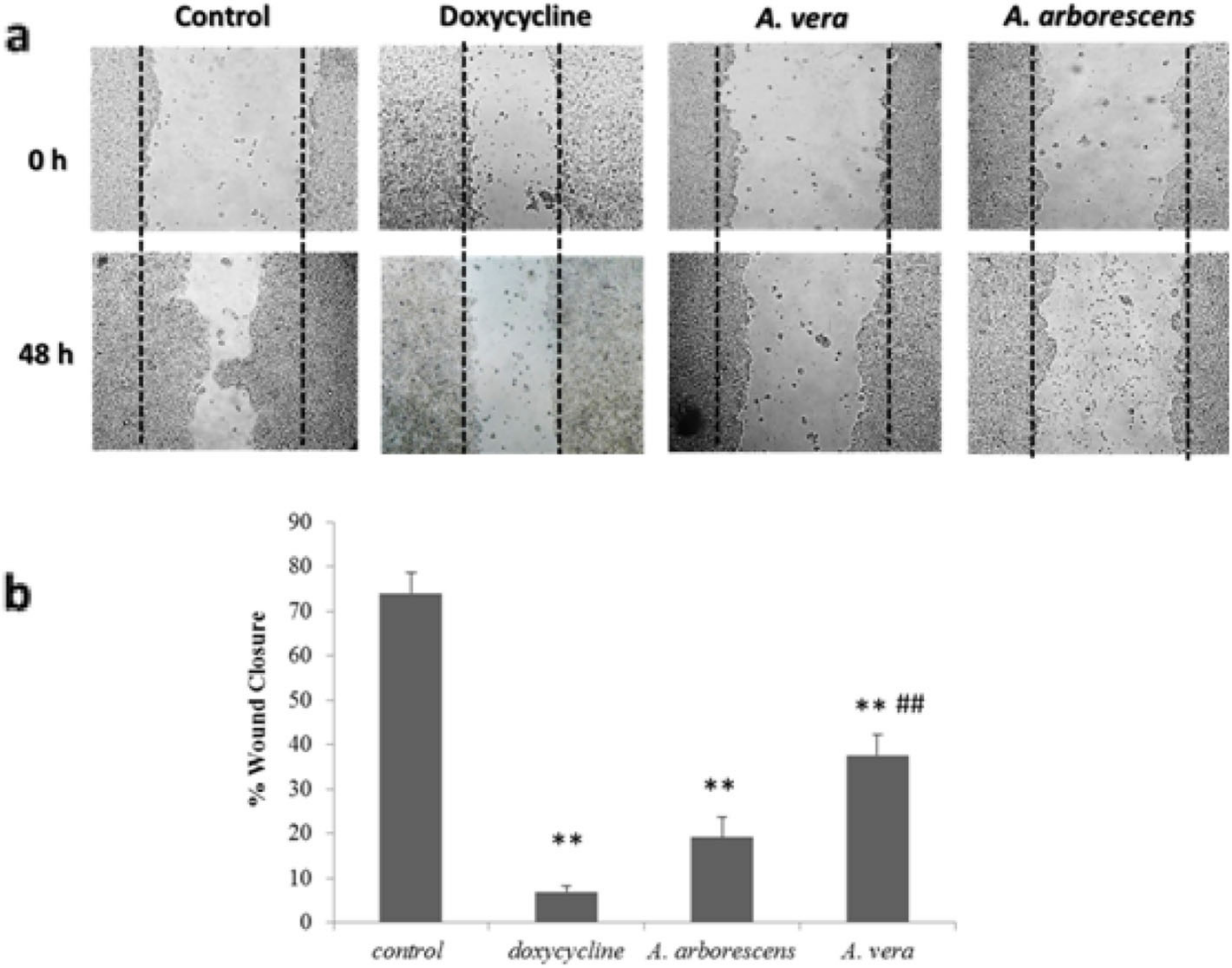
Wound healing assay on HT29 cells exposed to *A. arborescens* or *A. vera.* Extracts were prepared with 50% (v/v) MetOH (methanol). **a** Examples depicting cells at time 0 h and after 48 h of exposure. **b** Wound closure after a 48 h period cell exposure to *A. arborescens* or *A. vera* extractions with 50% (v/v) methanol. Doxycycline at a concentration of 40 μg/mL was used as a positive control for metalloproteinase inhibition [32]. *p < 0.05; **p < 0.001 when compared to positive controls; # *p* < 0.05; ## *p* < 0.001 when compared to doxycycline

Concerning the wound healing assay, we set out to characterize the migratory response of HT29 cells when exposed to the 50% (v/v) methanolic extractions of *A. arborescens* and *A. vera*. Since the EC50 concentration was around 25 μg/mL, this concentration was selected for the wound healing assays and the results are shown in Fig. 4. The percentage of wound closure of cells exposed to the *Aloe* extracts was compared to a positive control (deoxycycline at a concentration of 40 μg/mL), which presented a gap above 50% after 48 h.

Results indicated that all treatments induce high significant differences (*p* < 0.001) when compared to control. Contrasting to the cell proliferation assays, significant differences were observed between *A. arborescens* and *A. vera* extracts (*p* < 0.05). While *A. arborescens* exhibited a wound closure of 19%, *A. vera* was less effective in inhibiting cell invasiveness, with 37% of wound closure, which was significantly higher than deoxycycline. Under these conditions, *A. arborescens* yielded a cell viability of 45% and *A. vera* of 35% (data not shown).

### Aloe extracts inhibit MMP-9 and MMP-2

**Fig. 5:**
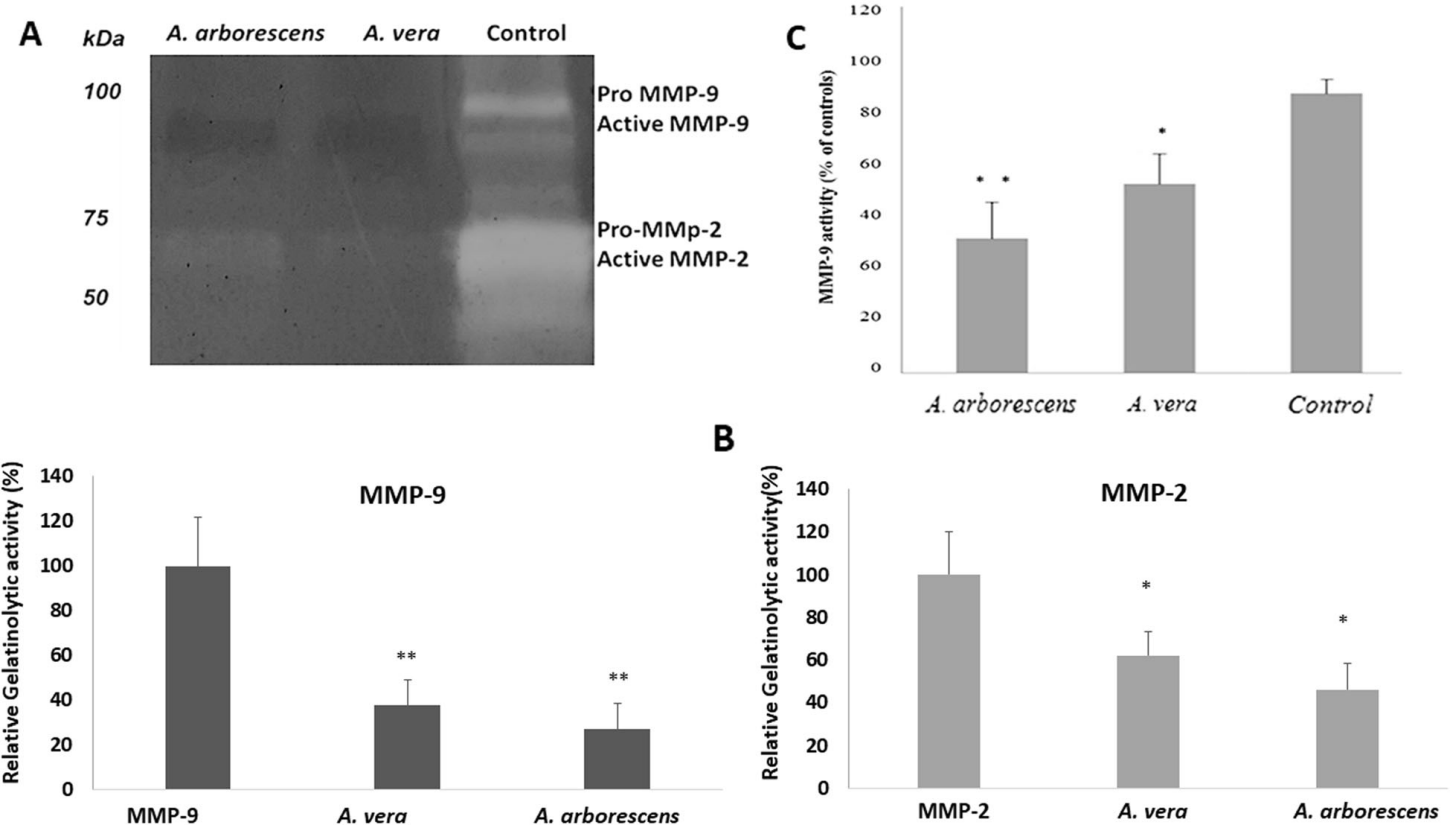
**a** Inhibition of gelatinolytic activity by *A. arborescens* and *A. vera* extracts. Extracellular HT29 cell media samples were subjected to a non-reducing gel electrophoresis (SDS-PAGE) in 12,5% /w/v) polyacrylamide gels supplemented with 0.2% (w/v) gelatin from bovine skin, type B. After renaturation, MMP-2 and MMP-9 activity was stimulated by incubation in reaction buffer (see material and methods) and the gel was stained for total protein. The presence of gelatinase activities was observed by clear bands against a dark blue background. **b** Relative activities of MMP-9 and MMP-2 bands calculated as a % of controls. **c** Gelatinolytic activities of commercial MMP-9 after exposure to *Aloe* methanolic extracts. MMP-9 was incubated in the presence of *A. arborescens* or *A. vera* extracts and its activity was quantified by the DQ-gelatin assay. Results are expressed as percentage of controls. Values are the mean of at least three replicate experiments ± SD. *p < 0.05, **p < 0.001

MMPs have been implicated in the migratory capacity of cancer cells due to its ability to degrade the extracellular matrix. Under this context, it was also our goal to test the activities of MMP-2 and MMP-9 in the extracellular media of the HT29 cells. Figure 5 shows the specific MMP-9 and MMP-2 gelatinolytic activity present in the extracellular media of HT29 cells, using zymography. In the gelatin zymography, white bands are indicative of non-inhibited MMP gelatinolytic activity, including that of the pro-enzyme and its active form. Concerning the MMPs, they are generally synthesized as zymogens (pro-MMPs), with their catalytic activity blocked by a cysteine switch and are only activated by its removal, through limited proteolysis. In the zymography assay, the pro-gelatinases similarly become active because they are denaturated by the SDS, therefore exposing the catalytic site (hence the slightly higher mass of the pro-enzymes in the zymography, which still maintain the short amino acid sequence of the cysteine switch). Consequently, there are two white bands for each MMP, as observed in the controls in Fig. 5 (a): gelatinase A or MMP-2 has a 72 kDa molecular mass, whereas gelatinase B or MMP-9 has 92 kDa.

When analyzing the lanes of the extracellular media exposed to the *Aloe* extracts, we can perceive that, after exposure to the *Aloe* extracts, MMP-9 and MMP-2 enzymatic activities were inhibited, in both forms, pro-enzyme and active form, with a more pronounced inhibitory specificity of both *Aloe* species towards MMP-9. In order to comprehend if the observed inhibitory activities were due to direct binding of *Aloe* extract components to MMP-9, we further tested their inhibitory activity concerning this enzyme. Figure 5 (c) illustrates the commercial MMP-9 (Sigma) activities in the presence of *A. vera* or *A. arborescens* extracts, expressed in percentage of controls. As observed in Fig. 5 (c), both *Aloe* extracts reduced MMP-9 activity directly, but this inhibition was higher for *A. arborescens* (*p* < 0.001) than for *A. vera* (*p* < 0.05).

## Discussion

Evidence has shown that certain bioactive phytochemicals are often present in more than one *Aloe* species [33], an observation that inevitably supports the species-substitution hypothesis in traditional medicine. Among the hundreds of identified *Aloe* species, there are two which stand out as the most common in European countries, *A. vera,* because of its huge popularity, and *A. arborescens*, because it is well adapted to the European climates and is widely spread across the public gardens as an ornamental plant. Although there are many differences between both species, such as morphological features, flower color, sap color, smell and other histological features, both species are often mistaken, as many media and anecdotal reports show. Although there are several articles highlighting the phytochemical composition of A. *vera*, much less is known about other species, like *A. arborescens*. In this work, we aimed to compare the potential anti-cancer and MMP-9 inhibitory activities between these two well-known *Aloe* species, *A. vera* and *A. arborescens*.

The literature describes several compounds in *A. vera* [33] which can be responsible for the various pharmacological activities previously described. The main phytochemical groups present in *A. vera* have been described as phenolic compounds (anthraquinones, anthrones), carbohydrates and proteins [3, 16, 33–36]. These different metabolites or phytochemicals may act individually, additively or in synergy to improve the plant’s health and its chances of survival in a given environment. Furthermore, it has been suggested that these combined actions of the phytochemicals usually tend to increase the bioactivity of the main medicinal constituents by influencing its assimilation in the body [15, 37]. Because of that, in this work, we not only aimed to compare the amounts of the potentially bioactive compounds in the two selected species, but we also wanted to select the best extraction procedure, that would allow the highest yield in these compounds. Therefore, we set out to quantify the major classes of bioactive compounds: proteins, total phenolic compounds and specifically anthraquinones, and total polysaccharides. When considering the whole leaf as an organ for phytochemical extraction, the possibility to combine several components has been suggested to potentiate its therapeutic activity [15, 37]. However, the choice of the extraction method by which the compounds are obtained was shown to be essential. Results here presented show that the extraction method not only influences the amount of bioactive component extracted but it also differs among species. Whilst for *A. vera*, for all compound’s classes except the proteins, the majority of the compounds were better extracted with 50% (v/v) methanol (phenolics and polysaccharides, with *p* < 0.05) for *A. arborescens* the highest yields were obtained in the 100% (v/v) methanol extraction. This suggests the presence of different compounds but more importantly, it arises the question of whether most literature using non-aqueous polar and non-polar solvents such as ethanol, acetone etc. are using the correct extractions to yield the better bioactivities with *A. vera*. This could be of significant importance since it is well-known that polysaccharides like acemannan were found responsible for the stimulation of the immune response in cancer scenarios, contributing to tumor weight reduction and the improvement of chemotherapy drugs [38, 39].

MMP-9 inhibitors (MMPIs) are considered metastasis deterrents and anti-angiogenic agents for colorectal cancer, and have also been proved to inhibit pre-cancerous states like colitis and other inflammatory bowel diseases [40]. Although many synthetic MMP inhibitors have been developed as potential anticancer drugs [40, 41], because of MMP’s ubiquity, most trials were hampered by dose-limiting toxicity, insufficient clinical benefits and lack of specificity. Natural, plant and food-based MMP9 inhibitors are currently more preferable to these inhibitors because of their lack of side effects and potentially higher specificity. In the last few years, a different perspective has emerged suggesting that using crude and/or standardized extracts as opposed to single compounds might be an advantage since each component has its major effect when acting synergistically with other components in the plant [14, 42, 43]. In this context, it also important to refer that there is an overall lack of consistency concerning the type of extractions to use. Some reports refer to aqueous extracts [15] whilst others use alcoholic or organic solvents such as methanol or acetone [16, 17]. Since the main phytochemical groups range from phenolic compounds to carbohydrates, proteins, organic and inorganic compounds as well as vitamins [3, 33–36], it becomes important to test different types of extractions to identify, which provide the highest amount of bioactive compounds, and allows the better synergy among them. After comparing the anticancer and MMP-9 inhibitory potential of two extracts from *A. vera* and A*. arborescens*, we selected the 50% (v/v) methanolic extractions for the remaining cancer cell assays. Interestingly, we also found that both Aloe extracts similarly reduce HT29 colon cancer cell proliferation in a dose-dependent manner. According to Lissoni et al. [44], this result was not surprising, as aloenin and other analogous molecules may be classified within the group of anthraquinonic and anthracenic substances, whose antiproliferative cytotoxic effects are widely known. Whilst the cell proliferation assay allows the assessment of growth inhibition, cell metabolism and overall cytotoxicity, the wound healing assay helps to assess the reaction of confluent cells in response to a disruption of cell-cell contacts. What generally occurs is a growth factors concentration increase at the wound margins stimulating proliferation and migration in order to close the opened gap [45], thus mimicking the metastatic process of cell migration. These two processes are particularly important when considering cancer development and have been associated to MMP activity, mainly MMP-9. Our results demonstrated significant differences between Aloe species when colon cancer cell migration was analysed. In fact, *A. vera* was less effective in inhibiting cell invasiveness than *A. arborescens*. Other reports using different plant extracts with known MMP-9 inhibitory activities [16, 17, 20, 30, 46–48] showed similar results and a known MMP-9 inhibitor, doxycycline in the same conditions induced similar reductions in cell migration [40]. These results suggest that although *A. vera* is the most used species and has been attributed numerous therapeutic benefits, regarding anti-cancer potential, it may not be as efficient as it has been described in reducing cancer cell migration. Indeed, even though there are several works highlighting the pytochemical composition of *A. vera*, much less is known about other species, like *A. arborescens*. Nonetheless *A. arborescens* has been the target of several studies in the last years, some of which associated to its anti-cancer properties [5, 44, 46, 49]. For example, *A. arborescens* was already proven to be effective in aiding chemotherapy when given orally at a dose of 10 mL thrice daily of a mixture consisting of 300 g of fresh leaves in 500 g of honey plus 40 mL of 40% (v/v) alcohol, either during or after chemotherapy [44]. This might be due to its capacity to inhibit tumor invasion and cell migration [30, 32].

Despite being effective in reducing cell migration, previous studies have already shown that some plant extracts, such as persimmon for example, can reduce cell migration but not through MMP-9 inhibition [47]. We therefore proceeded to evaluate if there was indeed MMP enzymatic inhibition.

MMP-2 was the first endopeptidase recognized to degrade collagens and to be related to the invasive and metastatic potential of cancer cells [45]. The active form of MMP-2 co-localizes with a pro-form of MMP-9 in various types of cancer, being able to activate it, consequently increasing tumor malignancy [50]. On the other hand, MMP-9 is linked to the promotion of metastization, angiogenesis and cell survival [23]. Since *A. vera* has been shown to concomitantly exhibit anti-inflammatory and anti-cancer activities, it appears reasonable to infer that targeting MMP-9 (a known key player in both conditions) could be one of Aloe’s biocomponents mode of action.

However, though there are many reports on the anticancer activities of *A. vera*, to our knowledge, there are no studies which correlate its anticancer activities with MMP-9 and MMP-2 enzymatic activity inhibition in cancer cells. Similarly, although there has been a considerable body of research associated to the effects of *A. vera* on cancer cells, very few use the whole extract, and even fewer have tested *A. arborescens*.

Our results demonstrated that both *A. vera* and *A. arborescens* extracts inhibited MMP-9 and MMP-2 enzymatic activities, especially towards MMP-9. Since MMP-9 is strongly associated to inflammation, wound healing and cancer migration [23, 51, 52], these results are more consistent to the anecdotal reports on *Aloe* bioactivities. A higher MMP-9 enzymatic inhibition can be of noteworthy importance because it has been considered that most MMPIs are non-specific, and this is, in turn, responsible for their generalized adverse side effects observed in most MMP studies. A more specific activity inhibition targeted exclusively to MMP-9, particularly in colon cancer, where this MMPI might act in situ, may be of significant potential to anticancer and anti-inflammatory approaches in the gastrointestinal diseases. Moreover, our results show that the detected inhibitory activities were due to direct binding of *Aloe* extract components to MMP-9, being this inhibition higher for *A. arborescens* than for *A. vera*. These results suggest that the higher MMP inhibitory activities in *A. arborescens* can be related to the higher inhibition of cell migration found for this species. Nevertheless, the percentage of activity reduction was not as high as the one observed in the zymographic analysis, where MMP-9’s activity was reduced more than 50%., suggesting that the *Aloe* extracts do not only inhibit directly this enzyme, but may also use alternative mechanisms. This is in agreement with the results obtained by Vijayalakshmi et al. [23] that showed *A. vera* extracts down regulating metalloproteinase expression, specifically MMP-9.

Regardless of having similar concentrations of various bioactive classes of compounds, *A. arborescens* seems to be a more effective cancer cell migration inhibitor than *A. vera*. This might be due to a larger concentration and/or greater number of bioactive compounds like anthraquinones present in *A. arborescens*, or to the presence of specific compounds not yet identified in this species. Even so, results show that in studies related to cancer prevention or therapy using *Aloe* species, *A. arborescens* should be considered as an effective alternative to *A. vera*. Additionally, our results also highlight the importance of the extraction procedure to obtain higher amounts of bioactive extracts, such as anthraquinones, phenolics and polysaccharides, particularly in *A. vera*.

## Conclusions

Our general results suggest specific important issues: 1) the genus *Aloe* is capable of inhibiting MMP-9 and MMP-2, although more efficiently MMP-9, 2) there are indeed differences among *Aloe* species, and 3) *A. vera* is possibly not the most efficient *Aloe* species. These facts allow us to ask important questions: is all the debate and misconception about *A. vera* being caused by erroneous uses of different species (more or less efficient, or more toxic than *A. vera*)? And are we missing out on not testing other *Aloe* species, in search for important bioactivities, which can efficiently be used in medical treatments? Either way, results here show that when considering the potential of *Aloe*, it is extremely important to take into consideration the type of extraction used, the species of *Aloe* and also their effects on individual MMPs.

## Data Availability

The data used and analysed in this study are available from the corresponding author on reasonable request.

## Abbreviations

DQ: Dye-quenched
MIC: Minimal inhibitory concentration
MMP: Matrix metalloproteinase
MMPI: Matrix metalloproteinase inhibitor
MTT: 3-(4,5-dimethylthiazol-2-yl)-2,5-diphenyltetrazolium bromide
PBS: Phosphate buffered saline
PVPP: Polyvinylpolypyrrolidone
